# Investigating risk of self-harm and suicide on anniversaries after bereavement by suicide and other causes: a Danish population-based self-controlled case series study

**DOI:** 10.1017/S2045796023000653

**Published:** 2023-08-08

**Authors:** Alexandra Pitman, Yanakan Logeswaran, Keltie McDonald, Julie Cerel, Gemma Lewis, Annette Erlangsen

**Affiliations:** 1 UCL Division of Psychiatry, University College London (UCL), London, UK; 2 Camden and Islington NHS Foundation Trust, St Pancras Hospital, London, UK; 3 College of Social Work, University of Kentucky, Lexington, KY, USA; 4 Danish Research Institute for Suicide Prevention, Psychiatric Centre Copenhagen, Hellerup, Copenhagen, Denmark; 5 Copenhagen Research Centre for Mental Health, Mental Health Center Copenhagen, Mental Health Services, Capital Region of Denmark, Hellerup, Copenhagen, Denmark; 6 Department of Mental Health, Johns Hopkins School of Public Health, Baltimore, MD, USA; 7 Centre for Mental Health Research, Research School of Population Health, The Australian National University, Canberra, Australia

**Keywords:** anniversaries and special events, attempted suicide, bereavement, suicide, time factors

## Abstract

**Aims:**

To investigate mechanisms of suicide risk in people bereaved by suicide, prompted by observations that bereaved people experience higher levels of distress around dates of emotional significance. We hypothesised that suicide-bereaved first-degree relatives and partners experience an increased risk of self-harm and suicide around dates of (i) anniversaries of the death and (ii) the deceased’s birthday, compared with intervening periods.

**Methods:**

We conducted a self-controlled case series study using national register data on all individuals living in Denmark from 1 January 1980 to 31 December 2016 and who were bereaved by the suicide of a first-degree relative or partner (spouse or cohabitee) during that period, and who had the outcome (any episode of self-harm or suicide) within 5 years and 6 weeks of the bereavement. We compared relative incidence of suicidal behaviour in (i) the first 30 days after bereavement and (ii) in the aggregated exposed periods (6 weeks either side of death anniversaries; 6 weeks either side of the deceased’s birthdays) to the reference (aggregated unexposed intervening periods). As an indirect comparison, we repeated these models in people bereaved by other causes.

**Results:**

We found no evidence of an elevated risk of suicidal behaviour during periods around anniversaries of a death or the deceased’s birthdays in people bereaved by suicide (adjusted incidence rate ratio [IRR_adj_] = 1.00; 95% confidence interval [CI] = 0.87–1.16) or other causes (IRR_adj_ = 1.04; 95% CI = 1.00–1.08) compared with intervening periods. Rates were elevated in the 30 days immediately after bereavement by other causes (IRR_adj_: 1.95, 95% CI: 1.77–2.22).

**Conclusions:**

Although people bereaved by suicide are at elevated risk of self-harm and suicide, our findings do not suggest that this risk is heightened around emotionally significant anniversaries. Bereavement care should be accessible at all points after a traumatic loss as needs will differ over the grief trajectory.

## Introduction

Suicide bereavement is linked to elevated risks of psychiatric illness (Pitman *et al.*, 2014), suicide attempt (Erlangsen *et al.*, 2017) and suicide (Pitman *et al.*, 2022), with likely genetic and environmental contributions (Pitman *et al.*, 2017a; Young *et al.*, 2012). Potential modifiable environmental risk factors for suicide after suicide bereavement include new or escalating depression or substance use, and social and emotional dimensions of suicide loss, such as stigma, eroded social support, loneliness and social modelling (O’Connor and Nock, 2014; Pitman *et al.*, 2014). Understanding the mechanisms by which risks of suicidal behaviour are increased after suicide loss could improve components of post-suicide emotional support (known as postvention). However, such mechanisms are under-investigated, perhaps explaining why existing postvention interventions lack evidence for reducing suicidality even while they are effective at reducing depression and anxiety (Andriessen *et al*., 2019; Linde *et al*., 2017; McDaid *et al*., 2008; Szumilas *et al*., 2011).

Identifying periods when risks of suicidal behaviour (i.e. self-harm and suicide) are increased after suicide loss is important when assessing risk and planning postvention, yet temporal risk factors for suicide are under-investigated (Nock *et al.*, 2019). Epidemiological evidence describes an increased suicide risk in the immediate aftermath of life events such as bereavement by any cause (Erlangsen *et al.*, 2004), discharge from psychiatric hospital (Qin and Nordentoft, 2005) or a cancer diagnosis (Henson *et al.*, 2019). However, most studies on the timing of suicide risk after bereavement investigate all-cause bereavement, identifying increases in suicide attempt or suicide in the initial aftermath (Ajdacic-Gross *et al.*, 2008; Barker *et al.*, 2014; Erlangsen *et al.*, 2004; Hiyoshi *et al.*, 2022) or around death anniversaries (Barker *et al.*, 2014; Bunch and Barraclough, 1971; Carr *et al.*, 2014; Chow, 2010; Hiyoshi *et al.*, 2022). Evidence suggests that the first week (Ajdacic-Gross *et al.*, 2008) and the first month (Ajdacic-Gross *et al.*, 2008; Hiyoshi *et al.*, 2022) after bereavement by any cause carries the greatest risk of suicide attempt or suicide. This is likely to represent an acute grief reaction (Oquendo *et al.*, 2014) and/or the worsening of pre-existing psychiatric illness in the context of carer burden or anticipatory bereavement (Kustanti *et al.*, 2022). The elevated suicide risk demonstrated over the first year after all-cause bereavement (Ajdacic-Gross *et al.*, 2008; Erlangsen *et al.*, 2004; Guldin *et al.*, 2017) may be linked to new onset or worsening depression or substance use and the longer-term impact of grief (including stigma and shame), loneliness and economic hardship (Stroebe *et al.*, 2007). The worsening of acute grief (Chow, 2010), mental disorders (Carr *et al.*, 2014), substance use (Hiyoshi *et al.*, 2022) and suicidal behaviour (Barker *et al.*, 2014; Hiyoshi *et al.*, 2022) observed at all-cause bereavement anniversaries, including during the lead-up period (Chow, 2010; Hiyoshi *et al.*, 2022), and the worsening of depressive symptoms around birthdays of a spouse deceased by any cause (Carr *et al.*, 2014), may represent temporal triggering of distress.

Anniversary reactions are defined as psychological, somatic and behavioural responses to a specific date, whether conscious or unconscious (Chow, 2010), and are observed in clinical practice (Renvoize *et al*., 1986; Gabriel, 1992), including where manifested as suicidal behaviour (Gabriel, 1992). Grief theories suggest that specific dates may revive thoughts of the deceased and reignite suppressed trauma (Chow, 2010), as consistent with psychodynamic concepts of repressed conflicts (Renvoize *et al*., 1986; Gabriel, 1992; Baker, 2001; Schechter *et al*., 2019). Yearning and preoccupation with the deceased around these times may create a strong desire for reunion and distress, contributing to suicidal thoughts (Young *et al.*, 2012). After suicide loss, the cognitive availability of suicide (and suicide methods) might also increase (Florentine *et al*., 2010; Biddle *et al*., 2012), contributing to acquired capability for suicide (O’Connor and Kirtley, 2018; Van Orden *et al.*, 2010).

No population-based studies have investigated anniversary effects after suicide loss specifically although exploratory findings in samples bereaved by all causes are suggestive of this (Barker *et al.*, 2014; Rostila *et al.*, 2015). Qualitative accounts describe a worsening of mood close to the anniversaries of a child’s suicide, their birthdays, and holidays, particularly in fathers (Entilli *et al.*, 2021), and the shared understanding among the suicide-bereaved that anniversaries are particularly difficult (Azorina *et al.*, 2019). We aimed to test whether key anniversaries after a suicide loss are associated with elevated rates of fatal and non-fatal self-harm compared with intervening time periods. Our hypothesis was that specific emotionally salient dates (death anniversaries, the deceased’s birthdays) are associated with increased rates of hospital-recorded self-harm (as a proxy for suicide attempt) and suicide.

## Methods

### Study design and data source

We applied a self-controlled case series (SCCS) design to registry data on the entire population of Danish-born individuals during 1980–2016, a method for investigating the association between a transient exposure and an adverse event (Petersen *et al.*, 2016; Whitaker *et al.*, 2006). In this approach, individuals act as their own controls, providing the advantage that all time-fixed observed and unobserved confounders (such as genetic factors or socio-economic status) are automatically accounted for in the analysis (Petersen *et al.*, 2016). Using a unique personal identification number assigned to all individuals at birth or first entry into Denmark (Erlangsen and Fedyszyn, 2015), we linked individual-level data from five nationwide population registers: the Civil Registration System (Pedersen, 2011), Registry of Social Pension and Income (Baadsgaard and Quitzau, 2011), Register of Causes of Death (Helweg-Larsen, 2011), National Hospital Register (Andersen *et al.*, 1999) and Psychiatric Central Research Register (Mors *et al.*, 2011).

### Participants

Cases were individuals of any age living in Denmark between January 1, 1980 and December 31, 2016 who had experienced the death of a first-degree relative (parent, offspring, sibling) or partner (spouses and cohabitees) over that period (regardless of where that death had occurred). First-degree relatives and partners were identified using information on family type, partner’s and household identification number, as noted in the Civil Registration System. We identified parents, children and siblings through a joint household identification number, irrespective of biological, step- or adoptive relations. Partners were defined by same- and opposite-sex marriages, civil partnerships and cohabiting couples, identified using a standard definition of cohabiting couples established within the Danish registers (Danmarks Statistik, 2022) (see Supplemental Methods 1), as per precedent (Erlangsen *et al.*, 2017). Relatives’ deaths were identified using the relevant International Classification of Diseases (ICD)-8 and ICD-10 codes from the Register of Causes of Death (Supplemental Methods 2).

**Figure 1.**
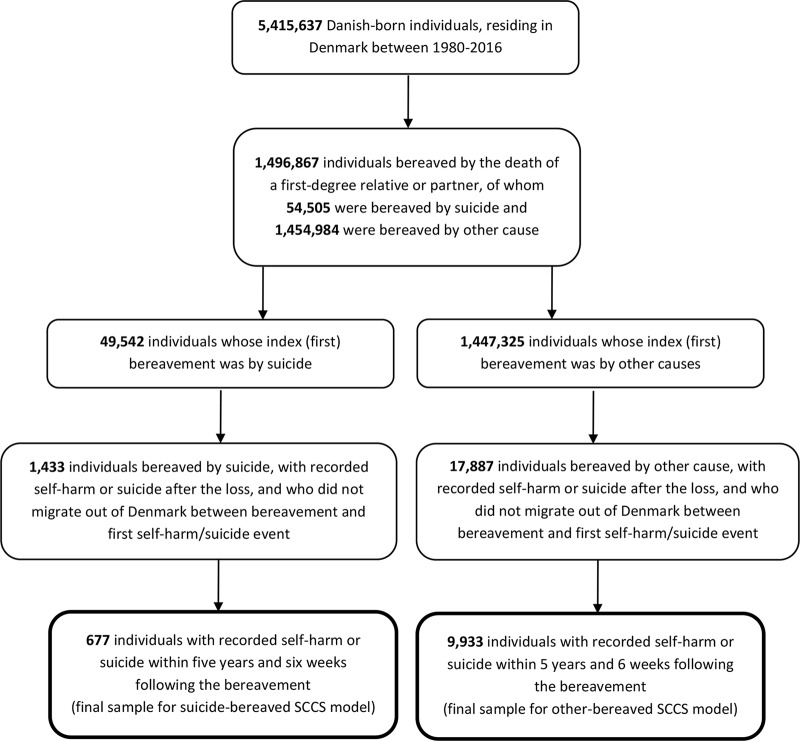
Flow of participants into SCCS models.

In an SCCS analysis, only individuals from the study population who experience the outcome and have both exposed and unexposed time periods during follow-up are included. This is to enable comparison of rates of the outcome between different time periods. We therefore included all those who had a recorded episode of self-harm or suicide within 5 years and 6 weeks of the bereavement, and who did not migrate out of Denmark between bereavement and the first self-harm/suicide event (Fig. 1). Among individuals who experienced multiple bereavements, we considered the first loss during the period of interest (regardless of relationship type) as the index bereavement.

We also wished to compare indirectly the risk estimates for suicide bereavement and non-suicide bereavement (capturing the emotional impact of bereavement *per se*). We therefore created two samples and modelled them separately for indirect comparison: (i) individuals bereaved by suicide and (ii) individuals bereaved by non-suicide death.

### Exposure

**Figure 2.**
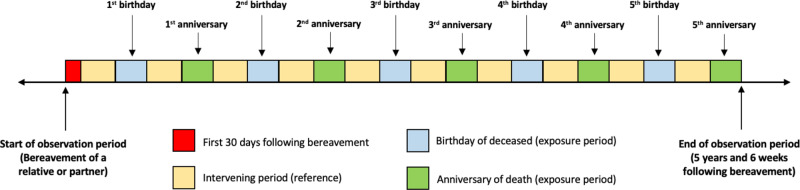
Timeline of a typical observation period for all SCCS models.

**Legend**: Each exposure period (period around the anniversary of the death; period around the birthday of the deceased) is 12 weeks, comprising the 6 weeks either side of the emotionally salient date. All are aggregated for the model.

Periods of emotional significance around bereavement anniversaries and birth anniversaries were defined as the 6 weeks either side of the bereavement anniversary and the 6 weeks either side of the deceased’s birthday, starting with the first anniversary or birthday after the loss. This period was chosen on clinical observations and research grounds to reflect anticipation of each event and its aftermath (Chow, 2010; Hiyoshi *et al.*, 2022). We restricted our exposed periods of interest to those in the first 5 years after the bereavement (Fig. 2) to ensure sufficient statistical power (due to a lower event rate in subsequent years), recognising that post-traumatic growth is reported at 3–5 years after a suicide (Levi-Belz, 2015).

We grouped together all exposure periods (five anniversaries and five birthdays) to create an *aggregated exposure period*. We defined (and aggregated) unexposed intervening (reference) periods as those post-dating the bereavement but pre-dating each anniversary/birthday period, those falling between anniversary/birthday periods and those between the last anniversary/birthday period and the end of follow-up.

We separated out the first 30 days after the loss for methodological reasons, given the recognised elevation in suicide risk in the immediate aftermath of all-cause bereavement (Ajdacic-Gross *et al.*, 2008; Hiyoshi *et al.*, 2022), which would otherwise inflate the event rate in the reference period.

### Outcomes

All self-harm episodes and suicide deaths over the period of interest were examined as outcomes, termed suicidal behaviour. Episodes of self-harm were identified as psychiatric and somatic hospital admissions for self-harm in the Psychiatric Central Research Register and the National Patient Register based on relevant ICD-8 (E950-E959) and ICD-10 (X60-X84) codes or where the reason for contact was recorded as “self-harm” (Supplemental Methods 2), as per the standard approach (Morthorst *et al.*, 2016). We restricted self-harm to episodes identified in secondary care, because this was more likely to identify medically severe cases in which high suicidal intent was assumed. Suicides were identified from the Register of Causes of Death using relevant ICD-8 (E950-E959) and ICD-10 codes (X60-X84, Y870). Any self-harm episode occurring within 7 days of a previously recorded self-harm episode was considered as the same event, using the date of the first attempt. Similarly, any suicide occurring within 7 days of a previous self-harm episode was considered as the same event (suicide), using the date of the suicide.

### Follow-up

Individuals were followed from the date of their index bereavement until censorship due to death by causes other than suicide, emigration, a second bereavement, the end of the observation period (i.e., 5 years and 6 weeks following bereavement) or December 31, 2016, whichever came first.

### Covariates

Models included one fixed (time-invariant) covariate and four time-varying covariates selected *a priori* (see Supplemental Methods 2 for detailed definitions) based on existing evidence supporting possible confounding.

Our fixed covariate was pre-bereavement self-harm rate in the 5 years prior to the index bereavement (limited by lead time available prior to bereavement). This acknowledged that past self-harm influences future risk of self-harm (Larkin *et al.*, 2014) and addressed the potential violation of the SCCS model assumption that recurrences of an event are independent (Petersen *et al.*, 2016).

Time-varying covariates were

- age (Fazel and Runeson, 2020)
- household income level (Fazel and Runeson, 2020)
- marital status (Kposowa, 2000)
- month at mid-point of each period: capturing seasonality of suicide in Denmark (Yip *et al.*, 2006).

### Statistical analysis

The socio-demographic and clinical characteristics of each sample were examined using descriptive analyses (Supplemental Methods 2 describes variable definitions).

Our SCCS models compared rates of suicidal behaviour during exposed and unexposed periods, commencing from the index bereavement, considering all episodes of self-harm and suicide during the follow-up period. Separate models were conducted for individuals bereaved by suicide and those bereaved by other causes.

Using fixed effects conditional Poisson regression, we estimated crude *incidence rate ratios* (*IRRs*) of suicidal behaviour and corresponding 95% confidence intervals (CIs), comparing (i) the first 30 days after bereavement and (ii) the aggregated exposure periods to our reference period (aggregated unexposed intervening periods). We adjusted IRRs for time-varying covariates and our fixed covariate, which in SCCS models are fitted as an interaction term (pre-bereavement self-harm rate × time period).

We used an adapted SCCS method for censored post-event exposures, developed to accommodate fatal outcomes by considering the *theoretical* risk of dying at any point during the study period (Farrington *et al*., 2009; Farrington *et al*., 2011; Petersen *et al.*, 2016; Whitaker *et al.*, 2006). This is an alternative to the standard SCCS method, which is conditional on the outcome event not affecting subsequent observations (Farrington, 2022). Fatal outcomes (e.g. suicide) would normally censor subsequent observations. However, the adapted approach accommodates fatal events by retaining individuals within the model even after a fatal outcome is recorded by extending the follow-up period until the end of the intended observation period. In practice, this means that the individual contributes time to the model after their death but no events until the end of that period, thereby avoiding truncating the person-time at risk (which would otherwise inflate the rate of an event during that interval relative to other intervals). We therefore followed up individuals who died by suicide until the end of the intended observation period (i.e. 5 years and 6 weeks after the bereavement).

We compared IRRs for individuals bereaved by suicide and those bereaved by other causes indirectly through visual inspection, as it was not possible to compare them directly.

### Sensitivity analyses

We ran SCCS models to test for potential biases introduced by use of an adapted SCCS approach, to account for potential acquired capability (O’Connor and Kirtley, 2018; Van Orden *et al.*, 2010) and to inspect yearly IRRs (see Supplemental Methods 3).

We ran a *post hoc* sensitivity analysis to inspect separate estimates for exposure to death anniversaries and to birth anniversaries.

Data preparation was performed in SAS software version 9.4 (SAS, 2003) and all analyses were conducted in Stata 17 software (StataCorp, 2021). We used a *p*-value threshold of 0.05 for all models.

## Results

### Sample characteristics

#### Suicide-bereaved

A total of 54,505 people (1.0%) experienced suicide bereavement over the period 1980–2016 in the entire Danish population of 5,415,637 individuals. Of these individuals, 49,542 people experienced suicide bereavement as their first (index) bereavement, following which 1,433 made a fatal or non-fatal suicide attempt prior to 2016. Of these, 677 had these outcomes recorded within 5 years and 6 weeks of the suicide bereavement and were included in our suicide-bereaved model (Table 1).

**Table 1.** Socio-demographic and clinical features of individuals recorded with a self-harm episode or suicide within 5 years and 6 weeks following bereavement by suicide or other causes

	Bereaved by suicide (*n* = 677)		Bereaved by other causes (*n* = 9,933)	
	*n*	%	*n*	%
**Socio-demographic variables**				
**Age^a^**	**Median**	**IQR**	**Median**	**IQR**
Age at bereavement	36	21−48	51	32−69
Age at censorship	41	26−53	56	37−74
**Sex**	***n***	**%**	***n***	**%**
Male	247	36.5	4,183	42.1
Female	430	63.5	5,750	57.9
**Household income level^b^**				
1 (lowest quartile)	91	13.4	693	7.0
2	109	16.1	1,152	11.6
3	82	12.1	1,152	11.6
4 (highest quartile)	38	5.6	436	4.4
Unknown	357	52.7	6,500	65.4
**Marital status^b^**				
Never married	313	46.2	2,967	29.9
Married/registered partnership	249	26.8	5,609	56.5
Divorced/dissolved partnership	111	16.4	1,263	12.7
Widowed/bereaved	4	0.6	93	0.9
Unknown	0	0.0	<3	–
**Kinship status of deceased**				
Parent	191	28.2	2,751	27.7
Child	59	8.7	396	4.0
Sibling	74	10.9	306	3.1
Partner	353	52.1	6,480	65.2
**Clinical variables**	**Median**	**Range**	**Median**	**Range**
Self-harm episodes during follow-up	1	1−8	1	1−29
	***n***	**%**	***n***	**%**
Suicide deaths during follow-up	145	21.4	2,613	26.3
Any pre-bereavement self-harm (binary measure)	151	22.3	1,684	17.0
	**Median**	**Range**	**Median**	**Range**
Pre-bereavement self-harm rate^c^	0.0	0.0−5.6	0.0	0.0−8.8
**Mental health disorders**				
Any listed below	201	29.7	2,834	28.5
PTSD	<3	–	18	0.2
Depression	54	8.0	992	10.0
Anxiety	26	3.8	212	2.1
Substance use	152	22.5	1,958	19.7
Severe mental illness	59	8.7	741	7.5
**Physical health disorders**				
Any listed below	45	6.7	1,157	11.7
Cardiovascular disease	18	2.7	645	6.5
Hypertension	7	1.0	178	1.8
Diabetes mellitus	12	1.8	202	2.0
COPD	10	1.5	274	2.8

IQR: interquartile range; PTSD: post-traumatic stress disorder; COPD: chronic obstructive pulmonary disease.

Any figures quoted as <3 indicate that cell size was below the threshold for reporting exact figures, as per the Statistics Denmark stipulations on protecting confidentiality.

a Time-varying covariate, using age at the start of each period.

b Time-varying covariates, values represented here are for year prior to bereavement (i.e. year prior to entry).

c Fixed covariate, covering the 5 years prior to bereavement (i.e. 5 years prior to entry), where lead time available.

#### Other-bereaved

A total of 1,454,984 people (26.9%) experienced bereavement by other causes over the same period. For 1,447,325 of these individuals, bereavement by other causes was their first (index) bereavement, following which 17,887 made a fatal or non-fatal suicide attempt prior to 2016. Of these, 9,933 had these outcomes recorded within 5 years and 6 weeks of the bereavement and were included in our other-bereaved model (Table 1).

Indirectly comparing the two samples, individuals bereaved by suicide were younger than individuals bereaved by other causes (median age at suicide bereavement = 36 [interquartile range (IQR) = 21–48] *versus* median age at other bereavement = 51 [IQR = 32–69]), and had a higher proportion who were women, never married or had a prior history of self-harm in the year prior to bereavement. Both groups had most frequently experienced the loss of a parent or partner and had a similar prevalence of past mental illness.

### Rates of suicidal behaviour around dates of emotional significance (main models)

We found no evidence of an increased rate of suicidal behaviour during emotionally salient time periods compared with intervening periods (Table 2) for suicide-bereaved (IRR_crude_: 0.97; 95% CI = 0.84–1.11; IRR_adj_ = 1.00; 95% CI = 0.87–1.16) or other-bereaved (IRR_crude_: 0.98; 95% CI = 0.94–1.01; IRR_adj_ = 1.04; 95% CI = 1.00–1.08) individuals.

**Table 2.** Incidence rate ratios for self-harm and suicide at any point over follow-up in (i) suicide-bereaved and (ii) other-bereaved individuals

Model			Unadjusted			Adjusted^b^		
Suicide-bereaved	Events	PDAR	IRR	95% CI	*p*-Value	IRR	95% CI	*p*-Value
Aggregated intervening periods (reference)	532	703,072	–	–	–	–	–	–
First 30 days after bereavement	31	20,987	**1.73**	**1.20−2.51**	**0.004**	1.49	0.98−2.25	0.062
Aggregated exposure periods^a^	348	482,204	0.97	0.84−1.11	0.631	1.00	0.87−1.16	0.969
**Other-bereaved**								
Aggregated intervening periods (reference)	7,253	10,134,706	–	–	–	–	–	–
First 30 days after bereavement	561	307,882	**2.22**	**2.03−2.42**	**<0.001**	**1.95**	**1.77−2.15**	**<0.001**
Aggregated exposure periods^a^	4,706	6,804,827	0.98	0.94−1.01	0.190	1.04	1.00−1.08	0.061

IRR: Incidence rate ratio; CI: confidence interval; PDAR: person-days at risk

a Exposure periods denote the 6 weeks either side of the bereavement anniversary and the 6 weeks either side of the deceased’s birthday, starting with the first anniversary or birthday after the loss, and ending 5 years and 6 weeks after the bereavement

b Adjusted for age, household income level, marital status, seasonality (all time-varying covariates) and pre-bereavement self-harm rate (fixed covariate).

There was an increased incidence rate in the 30 days following bereavement by other causes (IRR_crude_: 2.22, 95% CI: 2.03–2.42; IRR_adj_: 1.95, 95% CI: 1.77–2.22) but not following suicide bereavement (IRR_crude_: 1.73, 95% CI: 1.20–2.51; IRR_adj_: 1.49, 95% CI: 0.98–2.25).

We found no evidence to support an interaction between pre-bereavement self-harm rate and time period in either sample for this model (suicide-bereaved: *p* = 0.520; other-bereaved: *p* = 0.110).

### Sensitivity analyses

Findings did not differ substantially from those in our main models, justifying use of the adapted SCCS approach. Although not our focus in this study, some differences in magnitude and direction were observed in specific models for the initial 30 days post-bereavement. This identified uncertainty about whether risk of suicidal behaviour was elevated in this initial period (see Supplemental Results 2; Supplemental Tables 1–3).

Findings of *post hoc* sensitivity analyses for separate exposures (see Supplemental Results 2; Supplemental Tables 4 and 5), found that, in contrast to our main models, in relation to death anniversaries, the risk of suicidal behaviour for the suicide-bereaved group was elevated in the first 30 days after loss (although of borderline significance), thereby mirroring the elevated risk for the other-bereaved group. In relation to birth anniversaries, in contrast to our main models, the risk of suicidal behaviour for the other-bereaved group was elevated around birth anniversaries as well as in the first 30 days after loss (as opposed to solely the latter).

## Discussion

### Main findings

We found no evidence to support an increased risk of medically treated self-harm or suicide in suicide-bereaved first-degree relatives or partners (or those bereaved by other causes) around emotionally charged anniversaries. These negative findings may be due to an absence of any true effect or inadequate power. Given the upper limit of our confidence intervals, an effect estimate of a 16% elevated risk in the suicide-bereaved cannot be ruled out. Findings of sensitivity analyses suggest that the much greater sample size of the other-bereaved group may account for contrasting findings for the two samples. It is possible that heterogeneity obscured detection of elevated risk in subsamples of bereaved adults, such as those defined by gender (Bunch and Barraclough, 1971; Entilli *et al.*, 2021; Hiyoshi *et al.*, 2022; Rostila *et al.*, 2015), kinship (Barker *et al.*, 2014; Bunch and Barraclough, 1971; Entilli *et al.*, 2021; Hiyoshi *et al.*, 2022; Rostila *et al.*, 2015) or age group, those who identified strongly with the deceased (given the association of perceived closeness and psychopathology after suicide loss (Cerel *et al.*, 2016)), and/or those who experience complicated grief (given its association with suicidality (Mitchell *et al.*, 2005)). However, we lacked measures of closeness or grief and did not conduct interaction tests for age or sex due to their limited statistical power. It is also possible that reminders of the deceased are distributed throughout the year, augmenting distress at wedding anniversaries, religious festivals and holidays (Carr *et al.*, 2014; Entilli *et al.*, 2021), including anticipatory periods (Chow, 2010; Hiyoshi *et al.*, 2022), as well as around one’s own birthday (Ajdacic-Gross *et al*., 2012; Compassionate Friends, 2017) and major life events (exam success, parenthood) (Chater *et al.*, 2022). Anniversary effects may be manifested in other measures we lacked: hopelessness, suicidal thoughts or desire for reunion. Finally, risk may be specific to bereavement anniversaries but not birthdays, as with Swedish findings (Rostila *et al.*, 2015).

Although not our main hypothesis, the increased rate of suicidal behaviour in the first month after bereavement by other causes (but not suicide) was surprising given the stigmatising nature of suicide bereavement over other losses (Hanschmidt *et al.*, 2016), evidence supporting the greater emotional impact of suicide loss relative to other causes (Erlangsen *et al.*, 2017; Pitman *et al.*, 2014), and the comparative lack of support perceived immediately after suicide loss (Pitman *et al.*, 2017b, 2018b). However, this finding was not robust to sensitivity analyses, with estimates for this initial period varying in direction and magnitude for different models. The much larger sample size in the group bereaved by other causes could explain the contrasting findings.

### Findings in the context of other studies

To our knowledge, no other studies have tested hypotheses about suicide-related anniversary effects specifically after suicide loss. One previous population-based prospective study investigated this in Swedish adolescents bereaved by parental death from any cause, providing evidence to support an anniversary effect for risk of suicide and suicide attempt, but only in women at the first and second anniversary (Hiyoshi *et al.*, 2022). Our own analysis differed in combining all anniversary periods (to avoid multiple testing), yet sensitivity analyses identified elevated risks in the fifth year after a suicide bereavement, and in the fourth and fifth years after bereavement by other causes. These positive findings for selected years could explain the null finding in our main analysis considering 5 years together in a population of all kinships.

Our observation that suicide risk is elevated in the first month after non-suicide losses is consistent with Swiss evidence identifying the first week (and to a lesser degree the first month) after a spouse’s death by any cause as carrying the greatest risk of suicide (Ajdacic-Gross *et al.*, 2008), and Swedish evidence identifying an elevated risk of self-harm and suicide in the month of a bereavement by any cause (Hiyoshi *et al.*, 2022). This is also consistent with British survey evidence from suicide-bereaved and other-bereaved young adults, in which equal proportions (a third) identified the first month as the most difficult stage (Pitman *et al.*, 2017b). Qualitative accounts of suicide loss describe the early stages as the most challenging emotionally (Entilli *et al.*, 2021; Pitman *et al.*, 2018a; Ross *et al.*, 2018), relating this to an initial search for answers, difficulties making sense of the loss (Entilli *et al.*, 2021; Ross *et al.*, 2018), emerging awareness of stigma and lack of support (Pitman *et al.*, 2018a), followed (for some) by a process of meaning-making and post-traumatic growth (Entilli *et al.*, 2021; Ross *et al.*, 2018). Our sensitivity analyses demonstrate uncertainty over whether risk of suicidal behaviour is elevated in the initial aftermath of bereavement, but this was not our main hypothesis and warrants further investigation in a larger study. Nevertheless, suicide-bereaved individuals do express a perceived need for proactive support early after loss, remaining available thereafter (Dyregrov, 2011; Pitman *et al.*, 2018a).

### Strengths and limitations

We used longitudinal data on the entire population of Danish-born first-degree relatives/partners bereaved by suicide in Denmark since 1980, with no loss to follow-up. Use of registry data meant we avoided the selection biases and recall biases inherent to survey designs. The Danish registers are known to be reliable for psychiatric research because they provide completed records of all mental health conditions (including self-harm) diagnosed during inpatient episodes from 1969, as well as cause of death data from 1970. Data collection is therefore uniform regardless of exposure to bereavement, with no issues of recall bias. The validity of Danish cause of death data relies on coding by physicians certifying deaths, and this may be subject to individual differences and secular changes (Helweg-Larsen, 2011). However, the reliability of suicide classification in Denmark is judged to be strong compared with other Scandinavian registers (Tøllefsen *et al.*, 2015). The validity of admission dates recorded in the Psychiatric Central Research Register is also judged to be good (Mors *et al.*, 2011). The lack of recall bias inherent to use of Danish registry data was a particular advantage in this study given that our analysis was reliant on defining the objective timing of self-harm and suicide in relation to significant events. However, it is possible that some suicide attempts occurred a few weeks before a fatal outcome, with intervening medical treatment, thereby misclassifying the timing of the event for our purposes. The inclusion of a household variable in the Danish Civil Register meant that our analysis could use a broader definition of a couple than solely legal unions, thus reflecting family structure more accurately. However, the specific registry definition used does not include same-sex cohabiting couples. Other limitations of analysing routine Danish registry data were the under-ascertainment of medically severe self-harm due to lack of primary care data or secondary care outpatient data, and the under-ascertainment of episodes of medically severe self-harm in which no healthcare presentations were made.

Our use of the SCCS method was well-suited to the analysis of relatively rare outcomes (Farrington, 2022; Wijlaars *et al.*, 2013), with self-matching accounting for all time-fixed measured and unmeasured confounders (such as past psychopathology) (Farrington, 2022), and for the investigation of fine-grained time periods. SCCS study designs also generate more precise effect estimates than more traditional observational designs (Farrington *et al.*, 1996). Use of the adapted SCCS method meant we could avoid censoring individuals even after a fatal event, yet use sensitivity analyses to test for the effects of specific biases in design. Our analysis was not adjusted for hospital-diagnosed depression, which might influence risk of self-harm at an anniversary. This was because recording of depression is influenced by a self-harm event, thus violating an assumption of the SCCS approach (Petersen *et al.*, 2016). Our null findings might therefore be due to patients becoming more depressed around anniversaries and help-seeking, thereby preventing suicide. Our exposure periods accounted for almost half of each calendar year and were defined based on clinical consensus. While it is possible that findings might differ using tighter margins around anniversaries, these could exclude anticipatory and aftermath effects (Hiyoshi *et al.*, 2022). A shortcoming of the SCCS method is that it yields incidence rate ratios and not absolute measures of risk, making it difficult to contextualise the associations (Farrington, 2022). However, we provided indirect comparison to models for other-bereaved individuals. Our study included people bereaved by a range of kinship types, for reasons of power and representativeness. However, this heterogeneity may have obscured specific associations in specific kinship groups, as well as by sex and age, and we did not test these interactions for reasons of power. Finally, given cultural influences on suicidality, our findings from Danish-born individuals may only be generalizable to other Scandinavian countries. Such hypotheses need testing in other settings where large, longitudinal samples are available.

### Clinical and policy implications

Knowing the timecourse of suicide risk after suicide bereavement is important clinically in timing suicide prevention interventions. Despite clinical observations of increased distress on key anniversaries relating to suicide loss (Young *et al.*, 2012), acknowledgement of this in suicide support resources (Compassionate Friends, 2017) and previous evidence suggestive of elevated suicide risk on suicide anniversaries (Barker *et al.*, 2014; Rostila *et al.*, 2015), we did not observe increased self-harm or suicide risk at those points, whether for suicide or non-suicide bereavement. Observations of an increased risk of suicidal behaviour in the immediate aftermath of non-suicide losses (and possibly after suicide losses) suggest that early support is indicated. The uncertainties highlighted in our sensitivity analyses, the high frequency of deaths at first attempt, and complementary qualitative literature (Dyregrov, 2011; Pitman *et al.*, 2018b) suggest that proactive bereavement support should be targeted at all first-degree relatives and partners soon after any loss, with continued offers of support throughout the grief trajectory. The wider literature prompts practitioners in primary care, secondary care and voluntary sector support organisations to be aware of the possibilty of increased psychological distress (Carr *et al.*, 2014; Chow, 2010), substance misuse (Hiyoshi *et al.*, 2022) or suicidal behaviuor (Hiyoshi *et al.*, 2022) in bereaved individuals when approaching dates of emotional significance, as well as in the immediate aftermath. As suicide-bereaved individuals often prefer peer support over formal support (Dyregrov, 2011), it is important for friends and relatives to be aware of potentially difficult dates.

Trial evidence supports the potential for formal psychological support to reduce the risk of depression and anxiety, if not suicidality (Andriessen *et al*., 2019; Linde *et al*., 2017; McDaid *et al*., 2008; Szumilas *et al*., 2011), but the influence of timing of support has not been evaluated. Guidance on how to cope with emotionally salient anniversaries after suicide loss includes suggestions around honouring the deceased on those dates (Compassionate Friends, 2017). Parents interviewed after a child’s suicide described the importance of celebrating their deceased child’s birthdays to ensure continuing bonds, although found it harder to mark their death anniversary in a positive way (Ross *et al.*, 2018). Again, such approaches require evaluation.

## Conclusions

We found no evidence to support our hypothesis of elevated self-harm or suicide risk around dates of emotional significance (anniversaries or birthdays) after bereavement by suicide or other causes. We observed an increased risk in the immediate aftermath of a death by other causes, and uncertainty about whether this applied after suicide loss. Wider evidence supports an increase in psychological distress around emotionally salient bereavement anniversaries. This suggests that support should be offered proactively after any loss and that clinicians should be vigilant as to heightened distress immediately after bereavement and around key anniversaries. Our findings suggest a need for further research to explore the nature and timecourse of grief reactions, psychopathology and cognitions about suicide in the immediate aftermath of suicide loss and around dates of emotional significance.

## Data Availability

Danish registry data are available to researchers with appropriate affiliations on formal application to Statistics Denmark: https://www.dst.dk/en/TilSalg/Forskningsservice.

## Supplementary material

For supplementary material accompanying this paper visit http://doi.org/10.1017/S2045796023000653. click here to view supplementary material
