# Supplementary material for: Investigating risk of self-harm and suicide on anniversaries after bereavement by suicide and other causes: a Danish population-based self-controlled case series study
Source: Epidemiol Psychiatr Sci. 2023 Aug 8;32:e53. doi: 10.1017/S2045796023000653 (PMC10465319; doi:10.1017/S2045796023000653)
Supplement: Supplementary file 1 [file S2045796023000653sup001.pdf]

# Investigating risk of self-harm and suicide on anniversaries after bereavement by suicide and other causes: a Danish population-based self-controlled case series study

8 July 2023

## Supplemental Methods 1: Explanation of the Danish registers' definition of a couple (whether married or cohabiting)

Statistics Denmark have developed an established algorithm for identifying partners (married or cohabiting), which draws on information from the following covariates derived from the Civil Register: personal identification number, family type, partner's personal identification number, and household identification number, as recorded (since 1968) in the Civil Registration System (CRS). The personal identification numbers are known as CRS numbers.

A legally married/registered couple are defined as:

- Any married couple (different or same sex; joined through civil marriage or religious marriage) with or without children under 18 years living at home.

A cohabiting couple are defined as:

- Any cohabiting couple (identified through a record of joint children, via linkage of children to parents' respective identification numbers), with or without children under 18 years living at home.
- Any cohabiting couple (different sex; age difference under 15 years, no indication of being genetically related, only two adults in the household aged 16+ years), with or without children under 18 years living at home.

Note that this definition of cohabitees excludes same-sex cohabiting couples, but any definition that included same sex cohabiting couples would over-estimate this number due to the high likelihood of misclassification of same-sex roommates as couples.

Note that these definitions may also include any child under 18 living with a person who has been married to one of their biological parents. This is conditional on that step-parent not having remarried, in which case the connection can no longer be made between the child and its stepfather or stepmother.

For our study we defined a partner as a cohabitee or legal spouse, as per the above.

## Supplemental Methods 2: Definitions of variables used to describe characteristics of the sample

| Category | Definition | Population register |
|---|---|---|
| **Variables used for participant definition** | | |
| Suicide | ICD-8 codes E950-E959; ICD-10 codes X60-X84 and Y87.0 or where manner of death was recorded as 'suicide' | Register of Causes of Death [since 1980] |
| Other death | Any death recorded in the Causes of Death Registry except suicide | Register of Causes of Death [since 1980] |
| Kinship status of deceased | parent/child/sibling/partner | Civil Registration System [since 1980] |
| **Outcome (suicidal behaviour)** | | |
| Suicide | As above | As above |
| Self-harm[a] | psychiatric/somatic hospital admissions for self-harm based on ICD-8 code E950-E959 or ICD-10 code X60-X84 or where 'reason for contact' was listed as self-harm | Psychiatric Central Research Register [since 1969]<br><br>National Patient Register [since 1977] [b] |
| **Sociodemographic factors** | | |
| Age[†] | Continuous variable; measured at the start of each period | Civil Registration System [since 1980] |
| Household income level[†] | Quartiles: calculated as the total income within the household divided by the total number of adults living in the household, then categorised into quartiles based on national annual income averages i.e. relative to the whole Danish population. | Registry of Social Pension and Income [since 1980] |
| Marital status[†] | Categorical variable: unmarried; married; widowed; divorced/separated. Note that this variable did not include cohabitation status, as used in our main exposure definition, but was intended to capture the confounding effect of divorce. | Civil Registration System [since 1980] |
| | | |
| Sex | Male/female | Civil Registration System [since 1980] |
| **Psychiatric Disorders[c]** | | |
| PTSD | ICD-8 code 309.81<br>ICD-10 code F43.1 | Psychiatric Central Research Register [since 1969] |
| Depression | ICD-8 codes 296.09, 296.2, 298.0, 300.4<br>ICD-10 codes F32.0-F32.2, F32.8-F32.9, F33.0-F33.2, F33.4-F33.9 | Psychiatric Central Research Register [since 1969] |

| | | |
|---|---|---|
| Anxiety | ICD-8 codes 300.0, 300.2<br>ICD-10 codes F40, F41 | Psychiatric Central Research Register [since 1969] |
| Substance use | ICD-8 codes 291, 303, 304<br>ICD-10 codes F10-F19 (excluding F1x.0) [d] | Psychiatric Central Research Register [since 1969] |
| Severe mental illness[e] | ICD-8 codes 295, 296.89, 298.29-298.99, 299, 301.83, 296.1, 296.3, 298.1, 296.0 (excluding 296.09)<br>ICD-10 codes F20-F29, F30-F31, F32.3, F33.3 | Psychiatric Central Research Register [since 1969] |
| **Physical Disorders[f]** | | |
| Cardiovascular disease | ICD-8 codes 413, 410, 420, 425, 427.91, 427.93, 427.94, 427.09, 427.10, 427.11, 427.19, 427.99<br>ICD-10 codes I20-I22, I30, I42, I44, I46.0, I47.2, I48, I50 | National Patient Register [since 1977] [b] |
| COPD | ICD-8 codes 491-492, 518<br>ICD-10 codes J41-J44, J47 | National Patient Register [since 1977] [b] |
| Diabetes Mellitus | ICD-8 code 250<br>ICD-10 codes E105, E109, E111, E115, E119, E131, E135, E139, E141, E145, E149 | National Patient Register [since 1977] [b] |
| Hypertension | ICD-8 codes 400-404<br>ICD-10 codes I10-I13, I15 | National Patient Register [since 1977] [b] |

ICD-8= International Classification of Disease 8th Revision (used in Denmark from 1969-1993)
ICD-10=International Classification of Disease 10th Revision (used in Denmark from 1994-2016)Note that ICD-9 was never implemented in Denmark, hence the transition from ICD-8 to ICD-10.
PTSD: post-traumatic stress disorder; COPD: chronic obstructive pulmonary disease
[†]Time-varying covariate. Note that seasonality was the fourth time-varying covariates used, operationalised as month at mid-point of each period, but is not shown in this table.
[a] Linkage to relatives was via the personal identification number in the Civil Registration System [since 1980]. For simplicity in defining the exposure risk periods, we replaced all births and deaths that occurred on 29 February among relatives/partners/study participants with 28 February.
[b] The National Patient Register is sometimes termed the National Hospital Register.
[c] Applies to codes/diagnoses recorded on inpatient psychiatric admissions.
[d] Lowercase x denotes all possible values within the specified digit's diagnostic category
[e] Defined as psychotic disorders, manic episode, bipolar affective disorder, and depression with psychotic symptoms.
[f] Applies to codes/diagnoses recorded on inpatient medical admissions.

## Supplemental Methods 3: Sensitivity analyses

In our first sensitivity analysis we repeated our main models restricted to self-harm episodes only, to assess whether inclusion of fatal attempts might bias estimates.

Second, we revised our main models so that individuals were censored on the date of a suicide (rather than using the adapted SCCS design of retaining individuals in the model after a fatal event).

Third, we revised our main models so that individuals were censored at the end of the year in which they experienced a fatal event (effectively one year and six weeks based on model intervals; a modification of the adapted design).

Fourth, we considered only the first event (fatal or non-fatal) during the follow-up period. This took into the account the potential for a first suicide attempt to contribute to risk of subsequent suicide attempts due to reduced fearlessness (acquired capability).

Finally, we grouped together yearly exposure periods (e.g., first anniversary and first birthday, etc.) and presented IRRs for each year to explore patterns in the potency of exposure to emotionally salient exposure periods over the first to fifth years after bereavement. This was to ascertain whether surviving a difficult anniversary without attempting suicide might bolster confidence in being able to cope with subsequent anniversaries, thereby diminishing risk across subsequent years.

## Supplemental Results 1: Events by period in each group

During the 266 weeks of follow-up, 31 (4.6%) of eligible individuals bereaved by suicide were recorded as having a self-harm/suicide episode within 30 days of the loss (of which 13 were fatal), 297 (43.9%) were recorded so within six weeks either side of any death anniversary or deceased's birthday (of which 45 were fatal), and 422 (62.3%) were recorded so during unexposed intervening periods (of which 87 were fatal). Of the 145 (21.4%) who died by suicide within the observation period, 88% died on their first attempt (following the loss), 8% on their second attempt, and 4% on their third or more attempt.

During the 266 weeks of follow-up, 549 (5.5%) of eligible individuals bereaved by other causes were recorded as having a self-harm/suicide episode within 30 days of the loss (of which 158 were fatal), 4,095 (41.2%) were recorded so within six weeks either side of any death anniversary or deceased's birthday (of which 949 were fatal), and 6,145 (61.9%) were recorded so during the unexposed intervening periods (of which 1,521 were fatal). Of the 2,613 (26.3%) who died by suicide within the observation period, 89% died on their first attempt, 9% had had one previous attempt, and 2% had had more than one previous attempt.

## Supplemental Results 2: Sensitivity analyses

For our first sensitivity analysis of *self-harm episodes only*, we restricted our suicide-bereaved sample to 532 individuals (79% of total sample) and our other-bereaved sample to 7,320 individuals (73.7% of total sample). Characteristics of these subsamples were similar to those in the full samples apart from younger age at bereavement (**Supplemental Table 1**). Findings from these models restricted to *self-harm episodes only* were similar to those for our main models for both suicide-bereaved and other-bereaved individuals (**Supplemental Table 2**):

i.e. no evidence of an increased rate of suicide or self-harm during emotionally salient time periods. The elevated rate in other-bereaved individuals during the 30 days after bereavement was slightly attenuated.

Findings from our second sensitivity analysis, with individuals *censored at date of suicide* (i.e. after a fatal attempt) were also similar to those of our main models regarding emotionally salient time periods and the first 30 days for the suicide-bereaved (i.e. no elevated rate) (**Supplemental Table 2**). However, for people bereaved by other causes the association changed direction for the first 30 days after bereavement, whereby the rate was significantly reduced ($IRR_{adj}$: 0.87; 95% CI: 0.77-0.98). As most suicides did not occur within the first 30 days, censoring individuals at date of suicide truncated the person-time at risk, consequently inflating the rate of attempts in the reference period relative to the first 30 days. This sensitivity analysis demonstrates the bias introduced through censoring individuals on a fatal outcome, thereby supporting use of the adapted approach for our main model.

Findings from our third sensitivity analysis when *censoring individuals at the end of the year in which they experienced a fatal event* were similar to those of our main models, but the IRRs for the 30 days following bereavement by other causes were slightly attenuated, albeit remaining significantly elevated (**Supplemental Table 2**).

Findings from our fourth sensitivity analysis using models *considering only the first event* (fatal or non-fatal), to reduce the potential influence of acquired capability, were similar to those for our main models, with no increased rate of suicide or self-harm during emotionally salient time periods (**Supplemental Table 2**). This does not support the idea that acquired capability might differentially influence risk over the period of follow-up. We did observe an elevated rate of self-harm or suicide in the first 30 days after bereavement for the suicide-bereaved

($IRR_{adj}$: 1.61; 95% CI: 1.06-2.45) and an augmented estimate for the other-bereaved ($IRR_{adj}$: 2.08; 95% CI: 1.88-2.30), in contrast to our main models where this elevated rate was only observed in other-bereaved individuals. This demonstrates that SCCS models are appropriate for studying events where their recurrence is likely not independent. In this case, censoring individuals at their first event diminished the pool of those with potential outcomes (i.e. those yet to attempt), thereby inflating the rate in earlier intervals.

Finally, findings from our fifth sensitivity analysis when considering *each of the five years after the bereavement in turn*, showed an elevated risk of suicide or self-harm during anniversary periods in the fifth year after a suicide bereavement, and in the fourth and fifth years after bereavement by other causes (**Supplemental Table 3**). This did not support our prediction that surviving a difficult anniversary without attempting suicide might build resilience for subsequent anniversaries. However, we also acknowledged the potential for survivor bias and for Type I error given multiple testing.

## Supplemental Table 1: Characteristics of individuals in models for those bereaved by suicide and other causes who did not die by suicide prior to 2016 (first sensitivity analysis)

| | Suicide-bereaved model (n=532) | | Other-bereaved model (n=7,320) | |
|---|---|---|---|---|
| **Socio-demographic variables** | | | | |
| **Age[a]** | **median** | **IQR** | **median** | **IQR** |
| Age at bereavement | 32 | 20-43 | 44 | 28-64 |
| Age at censorship | 37 | 25-47 | 49 | 32-69 |
| **Sex** | **n** | **%** | **n** | **%** |
| Male | 166 | 31.2 | 2,656 | 36.3 |
| Female | 366 | 68.8 | 4,664 | 63.7 |
| **Household income level [b]** | | | | |
| 1 (lowest quartile) | 84 | 15.8 | 671 | 9.2 |
| 2 | 91 | 17.1 | 1,030 | 14.1 |
| 3 | 70 | 13.2 | 1,002 | 13.7 |
| 4 (highest quartile) | 32 | 6.0 | 376 | 5.1 |
| Unknown | 255 | 47.9 | 4,241 | 57.9 |
| **Marital status [b]** | | | | |
| Never married | 278 | 52.3 | 2,658 | 36.3 |
| Married/registered partnership | 169 | 30.1 | 3,566 | 48.7 |
| Divorced/dissolved partnership | 91 | 17.1 | 1,039 | 14.2 |
| Widowed/bereaved | 3 | 0.6 | 56 | 0.8 |
| Unknown | 0 | 0.0 | <3 | - |
| **Kinship status of deceased** | | | | |
| Parent | 170 | 32.0 | 2,468 | 33.7 |
| Child | 42 | 7.9 | 344 | 4.7 |
| Sibling | 68 | 12.8 | 285 | 3.9 |
| Partner | 252 | 47.4 | 4,223 | 57.7 |
| **Clinical variables** | | | | |
| | **median** | **range** | **median** | **range** |
| self-harm episodes during follow-up | 1 | 1-8 | 1 | 1-29 |
| | **n** | **%** | **n** | **%** |
| any pre-bereavement self-harm (binary measure) | 121 | 22.7 | 1,419 | 19.4 |
| | **median** | **IQR** | **median** | **IQR** |
| pre-bereavement self-harm rate [c] | 0.0 | 0.0-5.6 | 0.0 | 0.0-8.8 |
| **Mental health disorders** | **n** | **%** | **n** | **%** |
| Any listed below | 158 | 29.7 | 2,273 | 31.1 |
| PTSD | <3 | - | 16 | 0.2 |
| Depression | 39 | 7.3 | 727 | 9.9 |
| Anxiety | 22 | 4.1 | 175 | 2.4 |
| Substance use | 125 | 23.5 | 1,653 | 22.6 |
| Severe mental illness | 45 | 8.5 | 574 | 7.8 |
| **Physical health disorders** | | | | |
| Any listed below | 34 | 6.4 | 758 | 10.4 |
| Cardiovascular disease | 13 | 2.4 | 406 | 5.6 |
| Hypertension | 6 | 1.1 | 126 | 1.7 |
| Diabetes mellitus | 10 | 1.9 | 142 | 1.9 |
| COPD | 7 | 1.3 | 178 | 2.4 |

IQR: interquartile range; PTSD: post-traumatic stress disorder; COPD: chronic obstructive pulmonary disease

Any figures quoted as <3 indicate that cell size was below the threshold for reporting exact figures, as per the Statistics Denmark stipulations on protecting confidentiality.
[a] Time-varying covariates, using age at the start of each period.
[b] Time-varying covariates, values represented here are for year prior to bereavement (i.e. year prior to entry). Note that seasonality was the fourth time-varying covariates used, operationalised as month at mid-point of each period, but is not shown in this table.
[c] Fixed covariate, covering the five years prior to bereavement (i.e. five years prior to entry), where lead time available.

**Supplemental Table 2: Incidence rate ratios for self-harm at any point over follow-up in a) suicide-bereaved and b) other-bereaved individuals (sensitivity analyses)**

| **Suicide-bereaved** | **Main model** | | **Self-harm episodes only** | | **Censored at date of suicide†††** | | **Censored at end of the year of a fatal event** | | **First event (self-harm or suicide) only** | |
|---|---|---|---|---|---|---|---|---|---|---|
| **Unadjusted** | **IRR** | **95% CI** | **IRR** | **95% CI** | **IRR** | **95% CI** | **IRR** | **95% CI** | **IRR** | **95% CI** |
| Aggregated intervening periods (reference) | - | - | - | - | - | - | - | - | - | - |
| First 30 days after bereavement | **1.73**** | **1.20-2.51** | 1.11 | 0.67-1.83 | **0.56*** | **0.34-0.92** | 1.36 | 0.94-1.97 | **2.15***** | **1.47-3.14** |
| Aggregated exposure periods† | 0.97 | 0.84-1.11 | 1.00 | 0.86-1.16 | 1.03 | 0.89-1.18 | 0.97 | 0.84-1.11 | 0.91 | 0.77-1.07 |
| **Adjusted††** | | | | | | | | | | |
| Aggregated intervening periods (reference) | - | - | - | - | - | - | - | - | - | - |
| Aggregated exposure periods† | 1.00 | 0.87-1.16 | 1.02 | 0.87-1.21 | 1.01 | 0.87-1.17 | 0.96 | 0.83-1.11 | 0.98 | 0.82-1.16 |
| First 30 days after bereavement | 1.49 | 0.98-2.25 | 1.00 | 0.58-1.72 | 0.65 | 0.38-1.11 | 1.45 | 0.95-2.20 | **1.61*** | **1.06-2.45** |

| **Other-bereaved** | **Main model** | | **Self-harm episode only** | | **Censored at date of suicide** | | **Censored at end of the year of a fatal event** | | **First event (self-harm or suicide) only** | |
|---|---|---|---|---|---|---|---|---|---|---|
| **Unadjusted** | **IRR** | **95% CI** | **IRR** | **95% CI** | **IRR** | **95% CI** | **IRR** | **95% CI** | **IRR** | **95% CI** |
| Aggregated intervening periods (reference) | - | - | - | - | - | - | - | - | - | - |
| First 30 days after bereavement | **2.22***** | **2.03-2.42** | **1.86***** | 1.67-2.07 | **0.80***** | **0.72-0.90** | **1.65***** | **1.51-1.80** | **2.52***** | **2.30-2.76** |
| Aggregated exposure periods† | 0.98 | 0.94-1.01 | 1.00 | 0.95-1.04 | 1.04 | 1.00-1.08 | 0.98 | 0.94-1.01 | **0.95*** | 0.91-0.99 |
| **Adjusted††** | | | | | | | | | | |
| Aggregated intervening periods (reference) | - | - | - | - | - | - | - | - | - | - |
| First 30 days after bereavement | **1.95***** | **1.77-2.15** | **1.57***** | **1.39-1.76** | **0.87*** | **0.77-0.98** | **1.89***** | **1.71-2.08** | **2.08***** | **1.88-2.30** |
| Aggregated exposure periods† | 1.04 | 1.00-1.08 | 1.03 | 0.99-1.08 | 1.03 | 0.99-1.07 | 0.97 | 0.93-1.01 | 1.03 | 0.99-1.08 |

**Legend:** IRR: Incidence rate ratios; CI: confidence interval. *** $p$<0.001; ** $p$<0.01; * $p$<0.05.

†Exposure periods denote the six weeks either side of the bereavement anniversary and the six weeks either side of the deceased's birthday, starting with the first anniversary or birthday after the loss, and ending five years and six weeks after the bereavement.

††Adjusted for age, household income level, marital status, seasonality (all time-varying covariates), and pre-bereavement self-harm rate (fixed covariate).

†††This model is equivalent to the standard self-controlled case series (SCCS) method, a condition of which is that the outcome event should not affect subsequent observations. As one of our outcomes is fatal, this condition is not met in the standard model, which is why we used the adapted SCCS method in our main models.

**Supplemental Table 3: Incidence rate ratios for self-harm and suicide by annual risk periods at any point over follow-up in a) suicide-bereaved and b) other-bereaved individuals (sensitivity analyses).**

| **Model** | **Suicide-bereaved** | | | | | **Other-bereaved** | | | | |
|---|---|---|---|---|---|---|---|---|---|---|
| **Unadjusted** | **IRR** | **95% CI** | **p-value** | **Events** | **PDAR** | **IRR** | **95% CI** | **p-value** | **Events** | **PDAR** |
| Aggregated intervening periods (reference) | - | - | - | 532 | 705,521 | - | - | - | 7,253 | 10,134,706 |
| First 30 days after bereavement | **1.74** | **1.20-2.52** | **0.003** | 31 | 20,987 | **2.23** | **2.05-2.44** | **<0.001** | 561 | 307,882 |
| 1st year aggregated exposure periods† | 1.23 | 0.99-1.53 | 0.057 | 99 | 102,014 | **1.19** | **1.12-1.26** | **<0.001** | 1,343 | 1,480,513 |
| 2nd year aggregated exposure periods† | 1.08 | 0.86-1.37 | 0.500 | 81 | 98,827 | 1.03 | 0.96-1.10 | 0.403 | 1,054 | 1,419,436 |
| 3rd year aggregated exposure periods† | 0.80 | 0.61-1.06 | 0.121 | 57 | 96,562 | **0.92** | **0.86-0.99** | **0.027** | 883 | 1,369,303 |
| 4th year aggregated exposure periods† | **0.72** | **0.53-0.96** | **0.025** | 49 | 94,669 | **0.89** | **0.83-0.96** | **0.002** | 794 | 1,305,235 |
| 5th year aggregated exposure periods† | 0.94 | 0.72-1.23 | 0.656 | 62 | 91,985 | **0.76** | **0.70-0.83** | **<0.001** | 632 | 1,230,340 |
| **Adjusted††** | | | | | | | | | | |
| Aggregated intervening periods (reference) | - | - | - | | | - | - | - | | |
| First 30 days after bereavement | 1.45 | 0.95-2.22 | 0.084 | | | **1.92** | **1.74-2.12** | **<0.001** | | |
| 1st year aggregated exposure periods† | 0.93 | 0.73-1.20 | 0.591 | | | 1.02 | 0.95-1.08 | 0.627 | | |
| 2nd year aggregated exposure periods† | 1.00 | 0.78-1.29 | 0.997 | | | 1.00 | 0.93-1.07 | 0.919 | | |
| 3rd year aggregated exposure periods† | 0.93 | 0.69-1.25 | 0.638 | | | 1.02 | 0.94-1.09 | 0.676 | | |
| 4th year aggregated exposure periods† | 0.98 | 0.70-1.36 | 0.891 | | | **1.14** | **1.05-1.24** | **0.001** | | |
| 5th year aggregated exposure periods† | **1.54** | **1.11-2.14** | **0.009** | | | **1.11** | **1.01-1.23** | **0.027** | | |

**Legend:** IRR: Incidence rate ratios; CI: confidence interval; PDAR: person-days at risk.

†Exposure periods denote the six weeks either side of the bereavement anniversary and the six weeks either side of the deceased's birthday, starting with the first anniversary or birthday after the loss, and ending five years and six weeks after the bereavement.

††Adjusted for age, household income level, marital status, seasonality (all time-varying covariates), and pre-bereavement self-harm rate (fixed covariate).

**Supplemental Table 4: Incidence rate ratios for self-harm and suicide at any point over follow-up in a) suicide-bereaved and b) other-bereaved individuals for death anniversaries**

| **Model** | | | **Unadjusted** | | | **Adjusted**[††] | | |
|---|---|---|---|---|---|---|---|---|
| **Suicide-bereaved** | **Events** | **PDAR** | **IRR** | **95% CI** | **p-value** | **IRR** | **95% CI** | **p-value** |
| Aggregated intervening periods (reference) | 701 | 917,210 | - | - | - | - | - | - |
| First 30 days after bereavement | 31 | 20,987 | **1.71** | **1.19-2.47** | **0.004** | **1.52** | **1.00-2.31** | **0.049** |
| Aggregated exposure periods[†] | 179 | 268,066 | 0.89 | 0.75-1.05 | 0.154 | 0.94 | 0.78-1.13 | 0.515 |
| **Other-bereaved** | | | | | | | | |
| Aggregated intervening periods (reference) | 9,490 | 13,131,946 | - | - | - | - | - | - |
| First 30 days after bereavement | 561 | 307,882 | **2.20** | **2.02-2.40** | **<0.001** | **2.00** | **1.81-2.22** | **<0.001** |
| Aggregated exposure periods[†] | 2,469 | 3,807,587 | **0.92** | **0.88-0.96** | **<0.001** | 1.02 | 0.97-1.07 | 0.406 |

**Legend:** IRR: Incidence rate ratio; CI: confidence interval; PDAR: person-days at risk

[†]Exposure periods denote the six weeks either side of the bereavement anniversary and the six weeks either side of the deceased's birthday, starting with the first anniversary or birthday after the loss, and ending five years and six weeks after the bereavement

[††]Adjusted for age, household income level, marital status, seasonality (all time-varying covariates), and pre-bereavement self-harm rate (fixed covariate).

**Supplemental Table 5: Incidence rate ratios for self-harm and suicide at any point over follow-up in a) suicide-bereaved and b) other-bereaved individuals for birth anniversaries**

| **Model** | | | **Unadjusted** | | | **Adjusted††** | | |
|---|---|---|---|---|---|---|---|---|
| **Suicide-bereaved** | **Events** | **PDAR** | **IRR** | **95% CI** | **p-value** | **IRR** | **95% CI** | **p-value** |
| Aggregated intervening periods (reference) | 673 | 918,344 | - | - | - | - | - | - |
| First 30 days after bereavement | 31 | 20,987 | **1.78** | **1.23-2.57** | **0.002** | 1.48 | 0.97-2.24 | 0.066 |
| Aggregated exposure periods† | 207 | 266,932 | 1.06 | 0.90-1.23 | 0.489 | 1.10 | 0.93-1.30 | 0.249 |
| **Other-bereaved** | | | | | | | | |
| Aggregated intervening periods (reference) | 9,140 | 13,126,741 | - | - | - | - | - | - |
| First 30 days after bereavement | 561 | 307,882 | **2.27** | **2.08-2.47** | **<0.001** | **1.99** | **1.81-2.20** | **<0.001** |
| Aggregated exposure periods† | 2,819 | 3,812,792 | **1.06** | **1.01-1.10** | **0.009** | **1.09** | **1.05-1.14** | **<0.001** |

**Legend:** IRR: Incidence rate ratio; CI: confidence interval; PDAR: person-days at risk

†Exposure periods denote the six weeks either side of the bereavement anniversary and the six weeks either side of the deceased's birthday, starting with the first anniversary or birthday after the loss, and ending five years and six weeks after the bereavement

††Adjusted for age, household income level, marital status, seasonality (all time-varying covariates), and pre-bereavement self-harm rate (fixed covariate).
